# Resting-state electroencephalographic correlates of cognitive reserve: Moderating the age-related worsening in cognitive function

**DOI:** 10.3389/fnagi.2022.854928

**Published:** 2022-09-14

**Authors:** Ana Buján, Adriana Sampaio, Diego Pinal

**Affiliations:** Psychological Neuroscience Laboratory (PNL), Research Center in Psychology (CIPsi), School of Psychology, University of Minho, Braga, Portugal

**Keywords:** resting-state EEG, cognitive reserve, moderation, age-related decline, cognitive function, current source density, lagged-linear connectivity

## Abstract

This exploratory study aimed to investigate the resting-state electroencephalographic (rsEEG) correlates of the cognitive reserve from a life span perspective. Current source density (CSD) and lagged-linear connectivity (LLC) measures were assessed to this aim. We firstly explored the relationship between rsEEG measures for the different frequency bands and a socio-behavioral proxy of cognitive reserve, the Cognitive Reserve Index (CRI). Secondly, we applied moderation analyses to assess whether any of the correlated rsEEG measures showed a moderating role in the relationship between age and cognitive function. Moderate negative correlations were found between the CRI and occipital CSD of delta and beta 2. Moreover, inter- and intrahemispheric LLC measures were correlated with the CRI, showing a negative association with delta and positive associations with alpha 1, beta 1, and beta 2. Among those correlated measures, just two rsEEG variables were significant moderators of the relationship between age and cognition: occipital delta CSD and right hemispheric beta 2 LLC between occipital and limbic regions. The effect of age on cognitive performance was stronger for higher values of both measures. Therefore, lower values of occipital delta CSD and lower beta 2 LLC between right occipital and limbic regions might protect or compensate for the effects of age on cognition. Results of this exploratory study might be helpful to allocate more preventive efforts to curb the progression of cognitive decline in adults with less CR, possibly characterized by these rsEEG parameters at a neural level. However, given the exploratory nature of this study, more conclusive work on these rsEEG measures is needed to firmly establish their role in the cognition–age relationship, for example, verifying if these measures moderate the relationship between brain structure and cognition.

## Introduction

An aging population is rapidly increasing. In Europe, people aged 65 or over constituted 20.8% of the total population in 2021 (Eurostat, 2022). Although this increase in life expectancy is *a priori* a positive fact, age is the primary risk factor for developing a neurodegenerative disease and its associated cognitive impairment (Hou et al., 2019). In 2018, 50 million people were diagnosed with dementia worldwide (Patterson, 2018). Research into factors that can protect against cognitive impairment and its progression plays a crucial role in maintaining the quality of life and preventing dependence in older adults.

Different neuroprotective mechanisms have been proved to moderate the relationship between age and cognitive status. Indeed, for the last twenty years, much has been studied about the neural mechanisms of resilience to cognitive impairment in the aging brain, mainly in the context of neurocognitive syndromes such as Alzheimer’s disease (AD). Investigation of cognitively healthy older adults is also a fruitful avenue to provide information about the neural resilience mechanisms able to curb the onset or the progression of cognitive deficits as shown by the works of the Cambridge Centre for Ageing and Neuroscience (Cam-CAN) group (Chan et al., 2018; Borgeest et al., 2020). However, such studies on healthy aging are still scarce (see Bartrés-Faz and Arenaza-Urquijo, 2011, for a review). In this frame, resilience has been defined as a better-than-expected cognitive performance relative to the degree of pathology in a given individual; so, a way to cope with the effect of pathology on cognition (Arenaza-Urquijo and Vemuri, 2020). Resilience is a general term to refer to different neuroprotective mechanisms, including various reserve-related processes such as cognitive reserve, brain reserve, brain maintenance, or compensation (Stern et al., 2020). Among them, the most investigated entity has been cognitive reserve (CR), referring to an active process indexing the flexibility of cognitive and neural processes that helps to explain the differential vulnerability of cognitive function to age-related changes and/or to brain pathology (Barulli and Stern, 2013; Stern et al., 2020).

Quantifying CR is a matter of constant reviews and developments as it is challenging to assess it directly. Most measures are derived from socio-behavioral indexes used as indirect proxies, such as educational or occupational attainment, intelligence, leisure activities, and other lifetime experiences (Stern, 2002; Lojo-Seoane et al., 2014). Also, composite measures including different proxies of CR have been used to indirectly measure reserve (Nucci et al., 2012). However, all these measures do not tackle the whole variability behind CR (Marqués et al., 2016). Therefore, an effort to unravel the neural correlates underlying CR has been the target of several studies. In this context, CR is proposed to be supported by more adaptable functional brain processes that constitute a more direct and objective measure of CR than the socio-behavioral indexes mentioned above (Steffener and Stern, 2012).

In this vein, brain imaging techniques, both structural (magnetic resonance imaging - MRI) and functional [functional MRI (fMRI) and positron emission tomography (PET)], have been used to investigate the potential neural correlates of CR along the age-related cognitive continuum. Structural MRI has been mainly employed to search for anatomical correlates of brain reserve, considered the more genetic and passive component of such reserve (i.e., brain volume, number of neurons, and synapses… see Bartrés-Faz and Arenaza-Urquijo, 2011 for a review; Arenaza-Urquijo et al., 2013). An interesting approach from structural MRI is the development of residual measures of CR that have led to significant contributions in the field (Reed et al., 2010; Zahodne et al., 2013, 2015; Habeck et al., 2017; Lee et al., 2019). Regarding fMRI and PET, both are valuable tools for looking for functional correlates of cognitive reserve, i.e., brain networks. In general, these investigations have shown an inverse association between regional blood flow in fMRI and reserve measurements in healthy older adults during cognitive tasks, demonstrating increased neural efficiency in individuals with higher CR (Bartrés-Faz and Arenaza-Urquijo, 2011).

Resting-state fMRI is particularly useful when studying older adults since it avoids demanding tasks that may be confounded by potential cognitive or motor deficits (Hausman et al., 2020). Generic resting-state networks have been proposed as a promising measure of brain flexibility and CR, mainly through the study of the functional connectivity (FC) within and between brain networks (Bastin et al., 2012; Wook Yoo et al., 2015; Lindbergh et al., 2019). Further, this is considered as a more accurate approach than studying specific networks for a given task, providing that activated networks may be too specific and dependent on the precise brain regions involved in the task (Stern et al., 2020). Older age has been associated with weaker FC within brain networks compared to FC patterns in younger adults but with stronger functional connections between networks (Grady et al., 2016). Arenaza-Urquijo et al. (2013) found that education, as a proxy of CR, had a positive association with the FC between brain areas such as the anterior cingulate cortex and the hippocampus as well as the inferior frontal lobe, posterior cingulate cortex, and angular gyrus. As a common result, the FC between brain networks is increased for high CR participants both in healthy adults and adults with cognitive impairment (see Anthony and Lin, 2018 for a review).

Alternatively, neurophysiological measures through electroencephalography (EEG) and magnetoencephalography (MEG) have proven to be a promising, almost inexpensive method to study the CR neural correlates. (M)EEG is particularly useful, given its relatively non-invasive nature and high temporal resolution. Especially relevant in the context of FC, the oscillatory brain activity is thought to be a key index of the coordinated activity in long-range brain networks (Moezzi et al., 2019). Hence, synchronization of EEG oscillations at the same or different frequency bands between distant brain regions is considered as a mechanism promoting information flow between those regions (Siegel et al., 2012). Therefore, brain oscillations can be taken as an index of brain network organization and, then, suitable to research the neural mechanisms supporting CR.

Although the literature on the EEG correlates of CR in healthy aging is not as prolific as with other imaging techniques, several findings suggest that EEG is sensitive to the electrophysiological changes associated with various CR proxies (Fleck et al., 2019). Some studies have tried to associate different resting-state EEG and MEG measures with CR (see Šneidere et al., 2020 and Balart-Sánchez et al., 2021, for reviews). For instance, Grandy et al. (2013) found that individual alpha peak frequency was highly correlated with the general factor of intelligence but without differences between younger and older adults. Fleck et al. (2017), using a CR composite assembled with years of education and verbal intelligence scores, investigated the differences in FC as reflected by rsEEG coherence between older and younger people with high and low CR. They observed higher levels of CR were associated with greater overall brain coherence in older participants, but the opposite pattern in the younger ones. Babiloni et al. (2021, 2020b) have studied rsEEG and its association with educational attainment in three different samples: cognitively normal older adults, older adults with subjective memory complaints (SMC), and older adults with amnestic mild cognitive impairment (MCI). For older adults with SMC and negative amyloid PET, higher CR (i.e., education) was related to higher alpha rhythms in posterior areas. In those SMC participants with positive amyloid, higher CR was related to smaller posterior alpha rhythms (Babiloni et al., 2020b). Finally, comparing healthy older adults and patients with amnestic-MCI, Babiloni et al. (2021) showed that higher CR may be related to changes in rsEEG posterior alpha rhythms in healthy aging and MCI patients. They interpreted the observed results in terms of neuroprotective and compensatory mechanisms of CR. Some rsMEG studies have suggested specific oscillatory MEG signatures of CR. In particular, higher CR is related to higher gamma activity (Yang and Lin, 2020; Griffa et al., 2021), as well as to lower power in the delta band and higher alpha power for the oldest-old (Griffa et al., 2021). In addition, lower CR has been associated with positive gamma asymmetry in the occipital region (Yang and Lin, 2020).

Considering all the results provided by both imaging and (M)EEG studies, it seems that CR can be reliably identified at a neural level. Moreover, Steffener and Stern’s (2012) model establishes that CR can be derived either from socio-behavioral proxies or from neural measures. However, to the best of our knowledge, no studies have investigated that specific neural processes or networks can act as correlates of CR moderating the relationship between age and cognitive function.

The current perspectives regarding research on cognitive reserve have underlined the importance of studying aging following a life span approach to get reliable measures that reflect individual differences in brain structure and function built over the years (Arenaza-Urquijo and Vemuri, 2020; Stern et al., 2020). The reasoning behind it is that cognitive reserve-related variables collected at different points in the life span can predict cognitive function later in life (Steffener and Stern, 2012). Ideally, this approach should be longitudinal by measuring CR and cognitive function at different ages, from early to late adulthood. This could provide a range of different cognitive profiles that can be tied to age-related changes in cognitive reserve. So, chronological age seen from this life span approach can be taken as a measure of life course–related brain changes impacting cognitive outcomes. Therefore, in this study, we pursue to identify potential indices to propose more objective and neural-based correlates of CR through the rsEEG assessment in a broad age range sample (from 18 to 82 years old).

To achieve our main aim, rsEEG variables were derived from power spectral density and connectivity measures for the main EEG frequency bands in humans (delta, theta, alpha 1, alpha 2, beta 1, and beta 2) since previous studies have found differences between high and low CR participants in some of these parameters (Fleck et al., 2017, 2019; Babiloni et al., 2020b,^2021^). Further, based on the recommendations of Stern et al. (2020) for the study of CR, we implemented the following statistical plan: (1) bivariate correlation analyses between a composite socio-behavioral proxy [Cognitive Reserve Index (CRI); Nucci et al., 2012] and rsEEG variables, given that the expression of the neural variables underlying CR has to be associated with a socio-behavioral proxy of reserve; and, (2) moderation analyses with the rsEEG variables that correlated with the socio-behavioral proxy (i.e., CRI) as moderators of the relationship between age and cognitive status, since the brain processes or networks that underlie CR must moderate the effect of brain changes in cognition. In contrast with the recommendations by Stern et al. (2020), no structural measures of brain change were collected from the participants in this study. However, as we stated above, we adopted a life span approach from early to late adulthood, considering age as a proxy of life course–related brain changes, given its role as a risk factor that impacts cognitive outcomes (Collaboratory on Research Definitions for Reserve and Resilience in Cognitive Aging and Dementia, 2022).

Therefore, following the conceptual research model proposed by Steffener and Stern (2012), this study aimed to analyze resting-state EEG measures as putative neural-based correlates of cognitive reserve. In this model, cognitive reserve is operationalized by behavioral, cognitive, or neural measures that can help maintain cognitive performance by moderating its relationship with age. Due to the significant deviations from current recommendations on CR research and the paucity of studies using EEG as a measure of CR, we have conceived this study as highly exploratory, which precludes us from establishing specific hypotheses. Indeed, this study aims to generate hypotheses to be explored in future research.

## Materials and methods

### Participants

Advertisements and informative talks were used to aid in recruiting young and middle-aged adults among University of Minho staff and students, as well as healthy older adults among users from different cultural associations and day-care centers in Portugal’s North region. Data were collected from 79 volunteers, of whom 56 participants met the inclusion criteria and composed the final sample. Selected participants (age range between 18 and 82 years), thus, met the following criteria: (1) intact or well-corrected sensory function (self-reported); (2) perform independently in instrumental activities of daily living according to the Portuguese version of the Lawton and Brody scale (Lawton and Brody, 1969; Reis et al., 2012); and, (3) absence of significant neurocognitive impairment as assessed through the Mini-Mental State Examination (MMSE; Folstein et al., 1975). Note that cutoff scores were adapted according to the participants’ years of education as established in the Portuguese version of the MMSE (illiterate: ≤15; from 1 to 11 years of formal education: ≤22; more than 11 years of formal education: ≤27; Santana et al., 2016). The initial screening also ensured that participants had no history of stroke, transient ischemic attacks, head injury, Parkinson’s disease, or other neurological and psychiatric disorders. Participants who were taking psychoactive medications or medications for sleep promotion were excluded.

**FIGURE 1:**
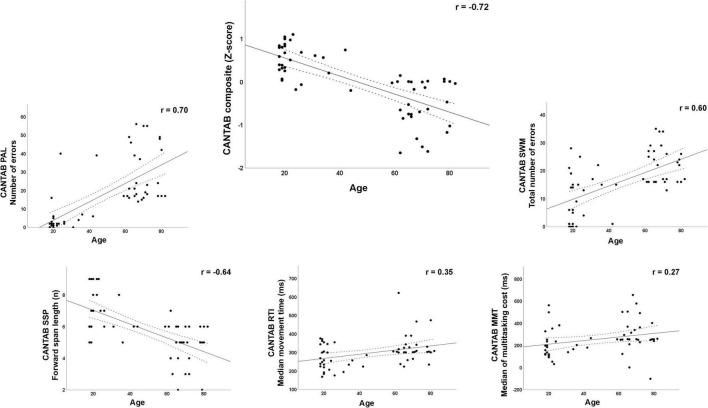
Scatterplots with Pearson’s *r* for the relationship between age and cognitive function measured through the CANTAB subtests and the composite score. PAL, Paired Associates Learning test; SSP, Spatial Span test; RTI, Reaction Time test; SWM, Spatial Working Memory test; MMT, Multitasking Test.

Table 1 summarizes the demographic (age, sex, and educational attainment), cognitive (MMSE score, CANTAB subtests, and CANTAB composite score), and CRI (scores for the total index and the three subindexes of the Cognitive Reserve Index Questionnaire, CRIq, see below for details) characteristics of the final sample. In addition, Supplementary Table 1 shows the correlations of age with the demographic and cognitive variables. Except for the CRI Education subindex and the total CRI, all variables were significantly correlated with age. The relationship between age and cognitive status, as reflected by the five CANTAB subtests used and the calculated composite score, is illustrated in the scatterplots in Figure 1.

**TABLE 1:** Demographic and cognitive characteristics [mean (SD)/ranges or %] for the total sample and the three groups of age.

Variables	Total sample (*n* = 56)	Young adults (*n* = 25)	Middle-age adults (*n* = 9)	Older adults (*n* = 22)
Age mean	46.50 (24.33)/18–82	21.32 (4.10)/18–35	54.56 (10.68)/36–64	71.82 (5.82)/65–82
Sex (% female)	87.50	92.00	88.90	81.80
Education years	11.42 (5.58)/0–24	14.74 (2.71)/12–23	12.56 (7.02)/4–24	7.18 (4.68)/0–17
MMSE score	28.86 (1.46)/23–30	29.48 (0.77)/27–30	28.78 (1.09)/27–30	28.18 (1.87)/23–30
CANTAB RTI	298.12 (79.08)/169–622	269.06 (67.75)/169–376.5	317.22 (118.91)/224–622	323.32 (62.60)/224–474.5
CANTAB PAL	17.13 (17.32)/0–56	4.00 (8.21)/0–40	24.22 (16.27)/6–49	29.14 (15.17)/14–56
CANTAB SSP	5.88 (1.66)/2–9	7.08 (1.26)/5–9	5.22 (1.30)/3–7	4.77 (1.27)/2–6
CANTAB SWM	16.11 (9.68)/0–35	10.20 (9.02)/0–28	17.89 (8.42)/1–29	22.09 (6.70)/13–35
CANTAB MTT	254.88 (148.85)/-101–656	211.50 (136.32)/32–563	259.44 (105.03)/124–503	302.32 (178.85)/-101–656
CANTAB composite	−1.65–1.10	0.55 (0.36)/-1.65–0.74	−0.25 (0.70)/-1.61–0.07	−0.52 (0.55)/-1.65–1.10
Total CRI	99.5 (14.11)/78–138	94.88 (9.37)/85–124	107.89 (15.99)/82–126	101.32 (16.29)/78–138
CRI education	98.98 (18.55)/77–151	98.72 (17.61)/80–151	105.78 (20.45)/80–134	96.50 (19.00)/77–140
CRI working activity	101.13 (12.24)/81–135	94.88 (3.28)/91–107	109.00 (15.72)/81–128	105.00 (14.05)/85–135
CRI Leisure	98.80 (11.49)/70–134	94.68 (4.40)/90–109	103.11 (13.82)/91–134	101.73 (14.61)/70–127

The study protocol was approved by the Institutional Review Board of the University of Minho (CE.CVS 095/2018) and conformed with the principles embodied in the Declaration of Helsinki. Before data collection, all participants were informed about the study and signed the corresponding informed consent form.

### Cognitive reserve and neuropsychological assessments

The CRIq (Nucci et al., 2012) was administered to assess CR. The CRIq comprehensively estimates the amount of CR accumulated by individuals throughout their lives by collecting information related to three typical proxies of CR, i.e., school (educational level), work (type and number of years of paid work), and leisure activities (how often the individual perform different activities such as reading newspapers, performing house chores, driving, using new technologies, social activities, and going to the cinema or the theater). An individual score or index is obtained for each of the three domains (CRI Education, CRI Working Activity, and CRI Leisure). Additionally, a total score (CRI) for the whole questionnaire can also be calculated and classified into one out of five categories: (1) low (CRI ≤ 70 points); (2) medium-low (CRI between 71 and 84 points); (3) average (CRI between 85 and 114 points); (4) medium-high (CRI between 115 and 129 points); and (5) high (CRI ≥ 130 points).

Regarding the cognitive function assessment, several subtests of the CANTAB (Cambridge Cognition, 2019) were applied. This computerized battery has demonstrated high sensitivity to detect changes in neuropsychological performance in the aging brain, both healthy and pathological (Junkkila et al., 2012; Juncos-Rabadán et al., 2014; Marsico et al., 2014). Although there are some studies devoted to the creation of normative data for specific CANTAB tests and establishing cutoff scores for cognitive impairment in the older population (Robbins et al., 1994; De Luca et al., 2003; Abbott et al., 2019), up-to-date there are no normative data for the Portuguese population. Our battery included tests to measure:

Psychomotor speed: the Reaction Time test (RTI), where the participant must hold a button at the bottom of the screen and react as soon as possible to release this bottom and select one out of five circles presented above whenever a yellow dot appears in one of them. The outcome measure in this study was the median of the movement time.

Memory: the Paired Associates Learning test (PAL) consists in learning the location of a series of patterns displayed behind boxes and, afterward, remembering where a specific pattern was initially located. The outcome measure used in this study was the total number of errors made by the participant. Also, the Spatial Span test (SSP), where a sequence of squares changing in color has to be remembered in the same order as they changed, with a progressive increase in the number of squares to be remembered. The outcome measure was the forward span length (the longest sequence successfully recalled).

Executive function: the Spatial Working Memory test (SWM) for which the participant must sequentially search for yellow “tokens” in each of several boxes. Planning and strategic thinking are needed as a box that had already contained a token cannot contain another one in the same trial. The selected outcome measure was the total number of errors (i.e., selecting boxes that have already been found to be empty and revisiting boxes that have already been found to contain a token). The Multitasking Test (MTT), where the participant has to indicate on which side of the screen an arrow appears or in which direction it is pointing according to a cue at the top of the screen (location vs. direction conditions). The task has single-task blocks, when the rule is consistent across trials, as well as multitasking blocks, when the cue changes from trial to trial in a randomized order. The outcome for this test was the median of the multitasking cost; that is, the difference between the median latency of response (from stimulus appearance to button press) during multitasking blocks and that of single-task blocks.

In order to have a single measure of cognition, a CANTAB cognitive composite score was derived by computing the z scores for each of the five subtests and averaging across the five scores.

### Electroencephalographic recordings and signal preprocessing

At least 3 min of EEG data were recorded from each participant while sitting comfortably and relaxed with eyes closed using an ActiveTwo Biosemi system (Biosemi, Amsterdam, the Netherlands) with 64 active electrodes inserted in an elastic cap according to the international 10-10 system. A Common Mode Sense (CMS) and Driven Right Leg (DRL) montage of two electrodes located around the vertex was used as a reference. Electrode offset was kept below 30 mV. EEG data were filtered online between 0.01 and 100 Hz and digitized at a sampling rate of 512 Hz. Simultaneously with EEG recordings, ocular movements were recorded with two electrodes located supra and infra-orbitally to the left eye and another pair situated at the lateral canthi of each eye.

After signal storage, rsEEG data from the 64 channels were preprocessed with EEGLab (Delorme and Makeig, 2004). First, the data were re-referenced to the nose tip, and a digital band-pass filter of 0.1--40 Hz was applied. Each participant’s EEG was visually inspected, and automated detection and correction of bad electrodes and bad data periods were applied using the Clean Raw Data EEGlab plug-in.^1^ An independent component analysis algorithm was then applied to extract independent components in the remaining data. ICLabel plug-in (Pion-Tonachini et al., 2019) was used to detect residual artifacts in the independent components, which were eventually removed from the data. Afterward, the removed channels were interpolated, data were re-referenced to an average reference, and 2 s epochs were created. Those epochs still presenting artifacts were removed according to different criteria (abnormal values of ± 100 μV, abnormal trends with a maximum slope of 75 μV, presence of improbable data and abnormal distribution with a single channel limit of 5 SDs, and abnormal spectral power in frequencies from 0 to 2 Hz (50 to −50 dB) and from 20 to 40 Hz (25 to −100 dB). Finally, as quality control, the power spectral density (PSD) was calculated through the p-welch function from the Signal Processing Toolbox, using Welch’s overlapped segment averaging estimator and windowed with a Hamming window. Those recordings with a minimum of 1 min of artifact-free data (Babiloni et al., 2020a) and standard spectra waveform in the eyes-closed resting condition were selected for posterior processing with exact low-resolution brain electromagnetic tomography (eLORETA; Pascual-Marqui, 2007a) to estimate the cortical sources of spectral density with a resolution of 0.5 Hz in six fixed bands: (1) delta (2–4 Hz); (2) theta (4.5–7.5 Hz); 3) alpha 1 (8–10 Hz); (4) alpha 2 (10.5–13 Hz); (5) beta 1 (13.5–20.5 Hz); and, (6) beta 2 (21–30 Hz). Despite being previously associated with CR in MEG studies, gamma activity (30.5–40 Hz) analyses are not included in this work. Source estimation of gamma rhythms has been shown to perform better when using MEG as compared to EEG signals (Mideksa et al., 2015). Further, gamma rhythms are difficult to record at the scalp (Nuñez and Srinivasan, 2010; Muthukumaraswamy, 2013), since this activity is typically highly artifacted by persistent EMG activity stemming from head and neck muscles’ tension (Goncharova et al., 2003; Whitham et al., 2008; Kropotov, 2016) as well as frequent microsaccades (Yuval-Greenberg et al., 2008; Yuval-Greenberg and Deouell, 2010) and even nasal breathing frequency (Tort et al., 2021). Nonetheless, the interested reader can find results from preliminary gamma analyses in the Supplementary Information Point 1.

The average rsEEG data length used to obtain the spectral values was 2.35 min, corresponding to a mean of 70 epochs per participant (SD: 0.39 min/11.64 epochs).

eLORETA is a genuine inverse solution (not merely a linear imaging method) with exact, zero error localization in the presence of measurement and structured biological noise (Pascual-Marqui, 2007a). Computations were made in a realistic head model (Fuchs et al., 2002), using the neuroanatomic Montreal Neurological Institute template (MNI152; Mazziotta et al., 2001), with the three-dimensional solution space restricted to cortical gray matter. The intracerebral volume was partitioned in 6,239 voxels at 5-mm spatial resolution. The first processing step in eLORETA was to compute EEG cross-spectra from the raw recordings using the 2-s epochs exported from EEGlab. Afterward, the cortical generators of surface oscillatory activity using the cross-spectra were computed. eLORETA solutions estimate current source density (CSD) values at x, y, and z vectors of any brain voxel able to predict EEG spectral power density at all scalp electrodes selecting the maximally smoothed solution among the possible infinite reconstructions of the active generators through a regularization procedure. This solution was normalized by the computation of the eLORETA CSD at each voxel averaged across all frequencies and all voxels. Finally, following the procedures by Babiloni et al. (2016), eLORETA solutions were averaged across all voxels in a given cortical macro-region of interest (ROI): frontal (Brodmann areas—BA −: 8, 9, 10, 11, 44, 45, 46, 47), central (BA: 1, 2, 3, 4, 6), parietal (BA: 5, 7, 30, 39, 40, 43), occipital (BA: 17, 18, 19), temporal (BA: 20, 21, 22, 37, 38, 41, 24), and limbic (BA: 31, 32, 33, 34, 35, 36) ROIs were considered. We estimated the current density of cortical sources as it provides a reference-free measurement with attenuated head volume conductor effects (Babiloni et al., 2016).

In addition, the eLORETA algorithm was also employed to obtain a measure of functional connectivity. Specifically, we calculated lagged-linear connectivity (LLC) as a measure of interdependence of rsEEG sources, given that it estimates linear inverse source connectivity while removing the artificially high zero-lag instantaneous interactions inherent to the low spatial resolution of the EEG tomography (Pascual-Marqui, 2007b). Although previous methods explore the connections between all possible pairs of locations, this “network approach” can test the joint dependence of several locations (Pascual-Marqui, 2007c). Hence, for each frequency band (i.e., delta, theta, alpha 1, alpha 2, beta 1, beta 2), the LLC was computed for the same six ROIs as the CSD (i.e., frontal, central, parietal, occipital, temporal, and limbic). We calculated both inter- and intrahemispheric LLC. The interhemispheric LLC was calculated between all voxels of the six ROIs of each hemisphere with the corresponding ones of the other hemisphere. For the intrahemispheric analysis, the LLC estimates were computed for all voxels of a particular ROI with all voxels of another ROI of the same hemisphere (i.e., frontal–central, frontal–parietal, frontal–temporal, frontal–occipital, frontal–limbic, central–parietal, central–temporal, central–occipital, central–limbic, parietal–temporal, parietal–occipital, parietal–limbic, temporal–occipital, temporal–limbic, and occipital–limbic for the right and the left hemispheres).

### Data analysis

Before the main statistical session, an exploratory analysis of the data was carried out. There were no missing data for the demographic and health variables, the CRIq, or the EEG variables. However, for the CANTAB subtests, some missing values were present (RTI, PAL, and SWM = 19.6% missing; SSP and MMT = 21.4% missing). These missing data were addressed by multiple imputations, creating 25 imputed datasets through a predictive mean matching procedure implemented in IBM SPSS (Version 27, IBM Corp, 2020).

In addition, given the percentage of missing data for the CANTAB subscales, to check that the multiple imputation process had worked properly, we compared the distribution of the variables from the original sample without imputations with the distribution of the same variables after the multiple imputation process. To that end, we conducted a one-way ANOVA, which pointed to the absence of significant mean differences between the two conditions for any of the CANTAB subscales (see Supplementary Information Point 2).

As expected, most of the rsEEG variables (i.e., eLORETA solutions) had a skewed distribution, and they were transformed to a logarithmic scale (log-10) (Babiloni et al., 2020b). No outliers^2^ were detected for CRIq, CANTAB, or rsEEG variables.

Descriptive and correlational analyses for demographic and neuropsychological variables were performed. Two types of inferential analyses were conducted. Firstly, the association between the proxy measure of CR and the rsEEG variables was tested through a series of bivariate correlations between CRI and each of the 252 rsEEG variables (36 CSD variables, 36 interhemispheric LLC variables, and 180 intrahemispheric LLC variables). The correlation coefficients are reported as Pearson’s *r* values. Given the exploratory nature of this study, instead of using the Bonferroni correction on associated *p* values to assess statistical significance, we have considered for further analyses only those correlations with at least a medium effect size (*r* ≥ 0.30; Cohen, 1988). Additional correlational analyses between rsEEG variables and cognitive outcomes (MMSE and CANTAB measures) as well as between rsEEG variables and CRI measures are out of the scope of this work but are publicly available at the Open Science Framework (OSF) register for this work (see data availability statement).

**FIGURE 2:**
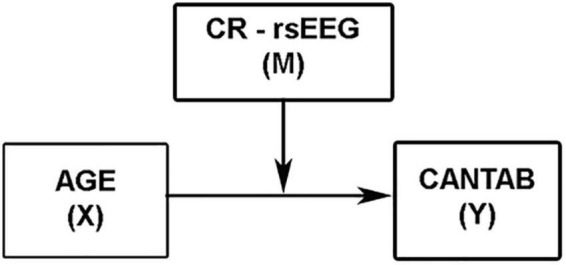
Conceptual representation of the simple moderation model.

Secondly, to address whether rsEEG variables can be used as direct measures of CR, a moderation analysis using the regression-based approach in Hayes’ (2017) PROCESS macro (Version 3.5) for IBM SPSS was conducted (Model 1; see Figure 2). Age was used as a continuous independent variable (X), and the CANTAB composite was introduced as the dependent variable (Y). Those rsEEG variables showing moderate effect size correlations with total CRI were entered as continuous moderator variables (M) and age × rsEEG variables as the interaction term (X × M). In addition, moderation analyses were also conducted with the CRI as well as each of the three CR subscales of the CRIq as moderators (M) of the relationship between age (X) and cognitive status (Y) to check whether these CR indices are reliable proxies of CR. Moreover, additional moderation analyses were performed using each of the five CANTAB subscales as dependent variables (Y). Sex was entered as a covariate in all the moderation analyses. Both the independent variable (age) and the moderators (rsEEG variables and CRIq scores) were mean-centered. Regarding the multiple comparisons problem in moderation analyses, the PROCESS macro adopts a multivariate linear regression that uses a bootstrapping methodology to calculate the confidence intervals for the regression coefficients. Although this solution does not entirely rule out type I errors, it provides an efficient way to ensure that the inferences are accurate (Hayes, 2017). Therefore, bootstrapped 95% confidence intervals (BootLLCI-BootULCI) are presented along with the regression coefficients.

To probe the existence of a significant interaction, an analysis of simple slopes is included in the PROCESS macro. Thus, the conditional effect of X (age) on Y (CANTAB composite score) at relatively low (16th percentile), moderate (50th percentile), and relatively high (84th percentile) values of M (rsEEG measures) were evaluated through this pick-a-point procedure (Hayes, 2017). In addition, to overcome the arbitrariness of the pick-a-point approach, the Johnson–Neyman (J-N) technique also implemented in PROCESS was used in this study to probe the significant moderation. This technique derives the values of the moderator to identify the “region of significance” of the effect of X on Y. These values of M demarcate the points along the continuum of M where the conditional effect of X on Y transitions between statistically significant and not significant levels (Hayes, 2017).

## Results

### Bivariate correlations between Cognitive Reserve Index and resting-state electroencephalographic variables

**FIGURE 3:**
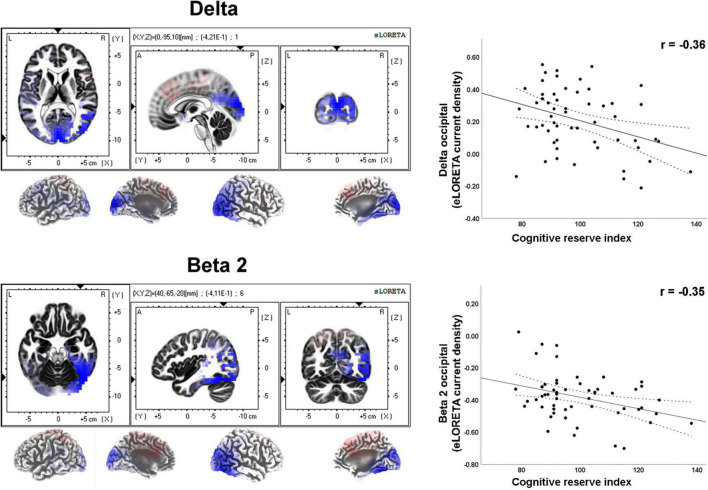
LORETA images and scatterplots with Pearson’s r for moderate correlations between CSD estimates and the Cognitive Reserve Index (*r* ≥ 0.30). The two correlations were negative (blue tones).

Moderate effect sizes were found for correlations between the CRI and several rsEEG CSD (i.e., normalized eLORETA solutions) for different frequency bands (Table 2). Negative correlations between CRI and delta and beta 2 CSD in the occipital region showed a moderate effect size (Figure 3).

**TABLE 2:** Pearson’s correlation coefficients and 95% confident intervals for moderate effect sizes of correlation between CRI and rsEEG variables.

rsEEG variables	Correlation coefficient	95% CI
**CSD**		
Delta occipital	–0.36	−0.60, −0.08
Beta 2 occipital	–0.35	−0.35, −0.13
**Interhemispherical LCC**		
Beta 1 temporal	0.40	0.12, 0.60
Beta 1 occipital	0.35	0.004, 0.58
**Right intrahemispherical LCC**		
Delta parietal–limbic	–0.44	−0.62, −0.22
Beta 1 frontal–occipital	0.37	0.08, 0.61
Beta 1 central–occipital	0.30	0.02, 0.51
Beta 2 occipital–limbic	0.30	0.08, 0.51
**Left intrahemispherical LCC**		
Alpha 1 frontal–temporal	0.30	−0.02, 0.55
Beta 1 frontal–temporal	0.30	0.05, 0.49
Beta 1 frontal–occipital	0.34	0.04, 0.55
Beta 1 parietal–temporal	0.34	0.05, 0.56
Beta 1 temporal–occipital	0.35	0.05, 0.57

**FIGURE 4:**
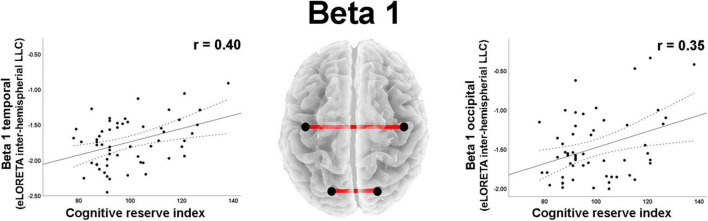
Graphs and scatterplots with Pearson’s *r* representing the connections (LLC measures) showing moderate size correlations between interhemispheric LLC estimates and the Cognitive Reserve Index (*r* ≥ 0.30). The two correlations were positive (red lines).

As regard to the LLC (i.e., connectivity) measures, moderate effect sizes were found for correlations between total CRI and inter- and intrahemispheric variables in different frequency bands (Table 2). Positive correlations were observed between CRI and beta 1 band interhemispheric LLC in temporal and occipital regions (Figure 4).

**FIGURE 5:**
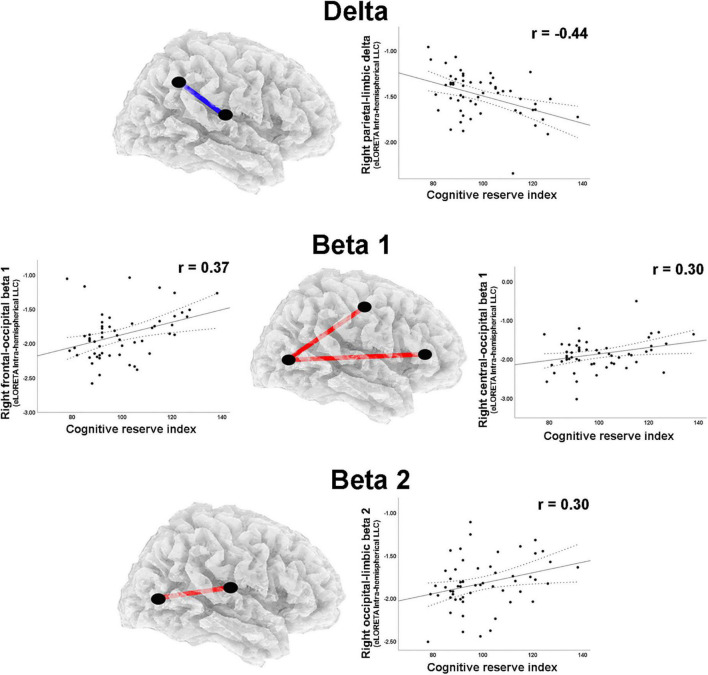
Graphs and scatterplots with Pearson’s r representing the connections (LLC measures) showing moderate size correlations between right intrahemispheric LLC estimates and the Cognitive Reserve Index (*r* ≥ 0.30). The correlation was negative for the delta band (blue line), whereas for beta 1 and beta 2 bands, the correlations were positive (red lines).

**FIGURE 6:**
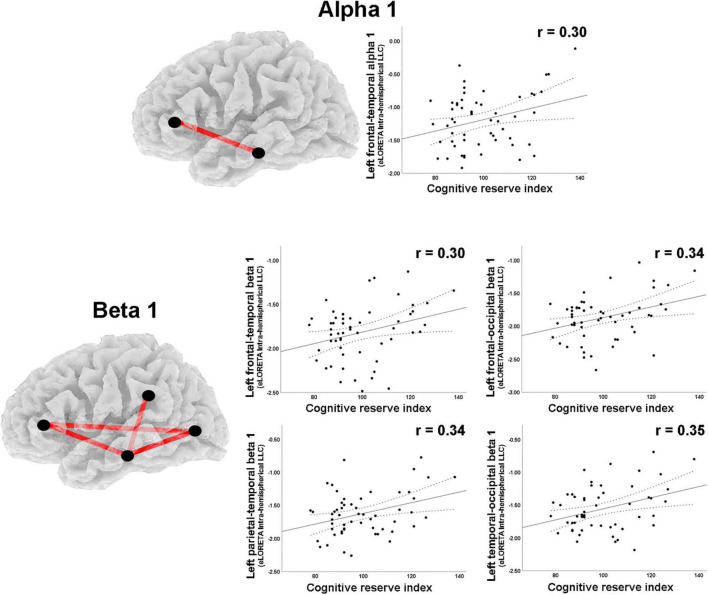
Graphs and scatterplots with Pearson’s r representing the connections (LLC measures) showing moderate size correlations between left intrahemispheric LLC estimates and the Cognitive Reserve Index (*r* ≥ 0.30). The significant correlations were positive for alpha 1 and beta 1 (red lines).

A moderate negative correlation was found between CRI and delta band intrahemispheric LLC involving parietal and limbic regions in the right hemisphere (Table 2). Positive correlations with moderate effect size were observed between CRI and intrahemispheric connections in the right hemisphere for: (1) beta 1 band LLC between frontal and occipital, as well as between central and occipital ROIs; (2) for beta 2 LLC between occipital and limbic ROIs (Table 2; Figure 5). Regarding the left hemisphere, positive correlations with moderate effect size were observed between CRI and: (1) alpha 1 LLC between frontal and temporal ROIs; (2) beta 2 LLC between frontal and temporal, frontal and occipital, parietal and temporal, as well as temporal and occipital ROIs (Table 2; Figure 6).

### Moderation analyses

For those rsEEG variables with a moderate size correlation with total CRI, moderation analyses were performed. Just two of the 13 variables significantly interacted with age to moderate cognitive status: the CSD of the delta band in the occipital ROI and the LLC of the beta 2 band in the right hemisphere between occipital and limbic regions.

**FIGURE 7:**
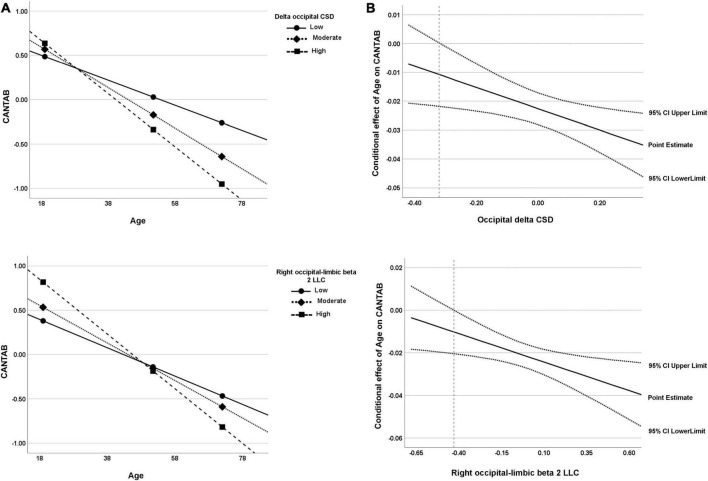
**(A)** Visual representation of the analysis of the simple slope for the two significant moderators of the relationship between age and cognitive function (CANTAB composite score). Data are plotted with regression-based slopes corresponding to the conditional effect of age on cognitive function at low (16th percentile), moderate (50th percentile), and relatively high (84th percentile) levels of estimated rsEEG measures (delta occipital CSD and right occipital–limbic beta 2 LLC). **(B)** Region of significance derived from the J-N technique. The region of significance is depicted as the values of the moderator corresponding to points where a conditional effect of 0 is outside of the confidence band (dotted lines). In other words, the value of the moderator for which a value of 0 is not within the confidence interval. The value from which the confidence region is outside 0 is -0.32 for occipital delta CSD and −0.43 for right occipital–limbic beta 2 LLC (dashed line). Both visual representations can be interpreted in the same way; for both measures, the higher the values, the higher the age-related cognitive worsening.

For the CSD of delta activity in the occipital region, the overall regression model was statistically significant, *R*^2^ = 0.61, *F*(4, 51) = 19.62, *p* < 0.001. As expected, a negative effect of Age on CANTAB score was significant, *b* = −0.02, *t*(51) = −8.25, 95% BootLLCI = −0.028-BootULCI = −0.017; *p* < 0.001, and although the effect of delta CSD was not significant, *b* = −0.67, *t*(51) = −1.96, BootLLCI = −1.27-BootULCI = 0.025; *p* = 0.06, the age × delta CSD interaction was significant, *b* = −0.04, *t*(51) = −2.47, BootLLCI = −0.065-BootULCI = −0.009; *p* < 0.05; Δ*R*^2^ = 0.05, *F*(1,51) = 6.59, indicating a moderator effect of delta CSD in the occipital region on the relationship between age and cognition. The covariate sex had no effect, *b* = 0.19, *t*(51) = 1.04, 95% BootLLCI = −0.25-BootULCI = 0.62; *p* = 0.30. The simple slope analysis indicated that the conditional effect of age on cognitive performance was statistically significant at any level of delta CSD in the occipital region (see Table 3 and Figure 7A). However, the J-N analyses revealed that below the delta CSD value of -0.32, the effect was not significant (Figure 7B). Nevertheless, just 9% of the cases (5 participants) were below that cutoff value. As shown in Figure 7A, the effect of age on CANTAB score is stronger for higher levels of delta CSD in the occipital region. Hence, the worsening of cognitive function with age increases as the CSD of delta at the occipital region also increases.

**TABLE 3:** Low (16th percentile), moderate (50th percentile), and relatively high (84th percentile) values of the significant moderators along with statistics.

Moderator	M values	Effect	SE	*t*	*P*	LLCI	ULCI
Occipital delta CSD	–0.230	–0.014	0.004	–3.191	0.002	–0.023	–0.005
	0.005	–0.023	0.003	–8.330	0.000	–0.028	–0.017
	0.197	–0.030	0.004	–7.785	0.000	0.038	–0.022
Right occipital–limbic beta 2 LLC	–0.214	–0.016	0.004	–4.629	0.000	–0.023	–0.009
	–0.019	–0.021	0.003	–7.688	0.000	–0.027	–0.016
	0.341	–0.031	0.005	–6.844	0.000	–0.040	–0.022

The intrahemispheric LLC between occipital and limbic regions for beta 2 activity in the right hemisphere was also a significant moderator of the effect of age on cognitive performance, *R*^2^ = 0.58, *F*(4, 51) = 17.83, *p* < 0.001. The effect of age was significant, *b* = −0.02, *t*(51) = −7.88, BootLLCI = −0.03-BootULCI = −0.02, *p* < 0.001, but the effect of beta 2 intrahemispheric LLC was not, *b* = 0.05, *t*(51) = 0.21, BootLLCI = −0.41-BootULCI = 0.53, *p* = 0.83. The interaction term between age and beta 2 intrahemispheric LLC was significant, *b* = −0.03, *t*(51) = −2.63, BootLLCI = −0.04-BootULCI = −0.005, *p* < 0.05; Δ*R*^2^ = 0.06, *F*(1,51) = 6.92. The covariate sex had no effect, *b* = 0.18, *t*(51) = 0.94, 95% BootLLCI = −0.19-BootULCI = 0.57; *p* = 0.35. Again, the conditional effect was significant for the three values of beta 2 LLC (see Table 3 and Figure 7A). The J-N analysis established a single cutoff value for significance at -0.43, being the lower values not significant (Figure 7B). However, these low values were only present for 7% of the data (4 participants). As in the case of delta CSD, the effect of age on CANTAB score is stronger for higher intrahemispheric LLC of beta 2 between occipital and limbic regions. Thus, showing an increasingly negative effect of age on cognitive performance as LLC of beta 2 between these right posterior regions increases.

Statistics for the rest of the non-significant moderation models are presented in Supplementary Table 2.

Moderation analyses with the CRIq indices (total CRI, CRI Education, CRI Working activities, and CRI Leisure) as moderators failed to reach statistical significance for each of the possible interactions with age (see statistics data in Supplementary Table 3). In addition, we also tested the moderation analyses using the different CANTAB subscales as dependent variables. Significant moderations were observed in the relationship between age and SSP performance (see Supplementary Table 4.1). Thus, such association was significantly moderated by: (1) occipital delta CSD, so the higher delta activity, the higher the age-related worsening of SSP performance; (2) the interhemispheric LLC for temporal beta 1 activity, so the stronger the connectivity, the lower the age-related worsening of SSP performance; and, (3) the right intrahemispheric LLC between frontal and occipital regions for beta 1 activity, so the stronger the connectivity, the lower the age-related worsening of SSP performance. Likewise, significant moderations were observed in the relationship between age and SWM performance (see Supplementary Table 4.2). Such association was significantly moderated by right beta 2 intrahemispheric LLC between occipital and limbic regions, so the stronger the connectivity, the higher the age-related worsening of SWM performance. The statistics for the non-significant models can be checked in the results files in the OSF repository.

## Discussion

This study aimed to analyze resting-state EEG measures (i.e., LORETA cortical sources’ current density and connectivity) as possible objective neural-based correlates of cognitive reserve following the model proposed by Steffener and Stern (2012). In this conceptual research model, cognitive reserve is operationalized by behavioral, cognitive, or neural measures that can help maintain cognitive performance by moderating its relationship with age. Therefore, we analyzed the relationship between rsEEG variables, CR, age, and cognitive performance in two statistical sessions: (1) Correlational analysis, to identify those rsEEG measures with at least a moderate size association with a socio-behavioral proxy of CR, the CR index (Nucci et al., 2012); and, (2) moderation analysis, to analyze whether any of those rsEEG variables that correlate with the CRI, were also able to moderate the effect of age on cognition. The results showed that many rsEEG measures presented at least a moderate correlation, either positive or negative, with the CRI. Still, just two of them were able to moderate the relationship between age and cognitive performance. Delta CSD in the occipital region and beta 2 LLC in the right hemisphere between occipital and limbic regions are suggested as possible correlates of cognitive reserve.

### Correlational analysis

Numerous neuroimaging studies have employed the correlational methodology to build potential associations between CR and structural or functional brain measures. In line with our results, in resting-state fMRI studies, higher CR has been associated not only with increased brain functions (i.e., stronger FC of anterior cingulate cortex with default mode network (DMN) regions, greater local efficiency, and clustering in cuneus and occipital regions) but also with decreased activity (i.e., lower metabolism in DMN and dorsal attention network regions) (see Anthony and Lin, 2018 for a review). The few rsEEG correlational studies performed up to date have shown no correlations at all (Amodio et al., 2017) or positive correlations (Grandy et al., 2013) with CR.

Despite the scarcity of rsEEG studies following the correlational approach, many studies have compared rsEEG variables between high and low CR groups. Sánchez-López et al. (2018) found less delta and theta activity in participants with high levels of physical activity, considered a protective factor for the cognitive function that has been sometimes proposed as a proxy of cognitive reserve (Dik et al., 2003; Fleck et al., 2019). In addition, an increase in delta oscillatory activity has been associated with cognitive dysfunction (Babiloni et al., 2006). In this line, in this study, an inverse correlation between the CSD of delta activity in the occipital region and the CRI was observed, so the higher the CRI, the lower the posterior delta activity. Thus, these low levels of delta might be considered as part of a neuroprotective or compensatory mechanism against cognitive decline. Further support for this assumption comes from the moderation analyses’ results, as discussed below.

A negative correlation between the CRI and the CSD of beta 2 was also observed in the occipital region. In some studies, decreased brain activity in higher CR participants has been related to more efficient neural mechanisms, even in resting-state. For example, Bastin et al. (2012) found that a higher degree of education and verbal intelligence was associated with less metabolism of the DMN and dorsal attention network regions, concluding this may be a mechanism to optimize resting-state brain functioning. Moreover, Rogala et al. (2020) have found that resting-state beta 2 activity averaged over all electrodes negatively correlated with behavioral performance. An increase in beta 2 activity for lower CRI values might likely reflect an unsuccessful compensatory mechanism reflecting higher and sustained cognitive effort in accordance with López et al. (2014). This result is probably pointing to a non-efficient organization of the functional networks. However, contrary to this tentative hypothesis, our connectivity results revealed a positive correlation between the CRI and the right intrahemispheric beta 2 LLC between occipital and limbic regions.

Inter- and intrahemispheric connectivity are considered measures of integration of the two cerebral hemispheres and hemispheric specialization, respectively. Further, they are paramount for good cognitive functioning (Liu et al., 2018; Chang et al., 2019). So, it seems coherent that higher CR was related to a more efficient integration and segregation between and within both hemispheres. In that sense, we observed that as the CRI increased, interhemispheric connectivity for beta 1 in temporal and occipital regions also increased. Similarly, intrahemispheric LLC in the right hemisphere for beta 1 between the frontal and occipital regions, and between central and occipital regions, were also positively correlated with CRI. In the left hemisphere, alpha 1 LLC between frontal and temporal regions and beta 1 LLC between frontal and temporal regions, frontal and occipital regions, and the temporal region and parietal and occipital regions also showed a positive correlation with CRI. These results are further in line with previous evidence of resting-state fMRI studies showing greater between network connectivity for high than low CR older adults (Anthony and Lin, 2018) as well as with the higher overall brain coherence in older high reserve participants than those with lower reserve (Fleck et al., 2017). In their 2017 study, Fleck and coworkers found a reversed relationship between overall brain coherence and CR when comparing the younger with the older adults in their sample (age range: 45–64 years of age), in a posterior study taking age as a continuous variable (range age 35–75) and using LLC instead of coherence, they did not found any significant effect of age (Fleck et al., 2019). However, these authors still observed important differences in LLC between high and low CR groups, with high social CR related to greater local and long-range LLC in theta and low alpha bands, and high cognitive CR associated with greater low alpha long-range LLC between the occipital and other cortical regions. The latter results are aligned with our results, indicating positive correlations between LLC measures and CR. As Fleck and coworkers, we believe that higher connectivity may reflect a neural compensation mechanism to cope with age-related brain structure and cognition declines. Furthermore, this results may indicate that such mechanisms are consistent across the adult life span, which fits within the current neurocognitive scaffolding theories of aging (Park and Reuter-Lorenz, 2009; Reuter-Lorenz and Park, 2014).

Nevertheless, as we noted above, increased FC is not always associated with better network functioning, both in resting-state and task-related activity (Buldú et al., 2011; Speer and Soldan, 2015). Our results are no exception, and we observed negative correlations between CRI and intrahemispheric LLC measures for delta, namely, the weaker the LLC for delta in the right hemisphere between parietal and limbic regions, the higher the CRI. Usually, such findings are interpreted in terms of better neural efficiency and organization of brain activity in high CR individuals compared with those with lower CR. Thus, our results may, as well as those of previous studies (see, for example, Fleck et al., 2017), indicate the coexistence of compensation mechanisms, as highlighted in the previous paragraph, together with better neural efficiency and reduced need for cognitive effort as reflected in the aforementioned negative correlations.

Alternatively, this complex pattern of positive and negative correlations between LLC and CRI may reflect differences in resting-state networks such as the DMN, dorsal attention network, and the frontoparietal network that have been previously shown to be anticorrelated in their activity (Fox et al., 2005). These resting-state functional networks have been associated with the different frequency bands studied in this work (see Mantini et al., 2007; Hlinka et al., 2010; Neuner et al., 2014; Gorantla et al., 2020). Therefore, future studies potentially combining fMRI and EEG methods are needed to explore whether there is a dissociated pattern of relationships between those anticorrelated resting state networks and the observed EEG connectivity correlates of CR.

### Moderation analysis

We performed moderation analyses with the rsEEG variables that showed a moderate correlation with CRI as moderators of the age-related prediction on cognitive scores (CANTAB composite). Out of the thirteen variables tested, just delta CSD in the occipital region and right beta 2 LLC between occipital and limbic regions were significant moderators of this relationship. More specifically, the negative relationship between age and cognitive performance observed in our sample was attenuated among people with less occipital delta activity and lower levels of right beta 2 LLC between occipital and limbic regions. As a tentative hypothesis, this might mean that CR could be manifested as a more efficient network with weaker activation. People with lower CR probably showed intensified activations as an attempted, although unsuccessful, compensatory mechanism.

As mentioned above, an increase in delta activity, especially in posterior areas, has been associated with cognitive impairment and reported in some pathological conditions such as AD (Babiloni et al., 2004, 2006; Benwell et al., 2020), strokes (Cassidy et al., 2020), vascular dementia (Babiloni et al., 2006; van Straaten et al., 2012), and Parkinson’s disease with cognitive impairment (Caviness et al., 2011; Fonseca et al., 2013). Therefore, this mechanism is probably related to a derangement in the functional thalamocortical connectivity due to neuropathology or neuroinflammatory processes (Babiloni et al., 2018). Further, resting-state delta CSD might be used as an objective index of CR since the available evidence, including our results, seems to confirm that higher delta activity could reflect cognitive vulnerability. In addition, this result is also in agreement with the results of Griffa et al. (2021) for the oldest-old participants, showing lower delta activity in a high compared to a low CR group.

A more paradoxical result was the moderation effect of the beta 2 LLC between occipital and limbic regions in the right hemisphere. The moderation analysis showed a more negative impact of age on the cognitive score as the beta 2 LLC increased, contrary to the positive correlation between occipital CSD in this frequency and CRI. This contradictory pattern seems to be due to the inclusion of the factor age and the interaction term in the moderation analyses. The effect of beta 2 LLC on CANTAB score was positive in the regression model (i.e., the higher the beta 2 LLC, the higher the CANTAB score), but when interacting with age, the effect turned negative. Although this relationship seemed the inverse at younger ages (i.e., better CANTAB scores at higher values of beta 2 LLC), as age increased, higher levels of beta 2 LLC seemed to lead to a worsened cognitive function. There are well-documented age-related differences in rsEEG variables in the literature, even in the field of CR. For example, Fleck et al. (2017) found higher coherence during eyes-closed for older adults (i.e., adults between 59 and 65 years of age) with high CR than for those with low CR, whereas the opposite pattern was found for their younger participants (i.e., adults between 45 and 58 years of age).

A negative correlation between beta 2 activity and behavioral performance has been previously observed by Rogala et al. (2020). They consider it an electrophysiological signature of the strength of long-range frontoparietal and fronto-occipital connections. In accordance with our tentative hypothesis regarding the CSD of beta 2, Rogala et al. (2020) suggested that weaker beta 2 oscillations in long-range networks might represent an enhanced capacity of network reconfiguration and, then, higher efficiency. Moreover, Santarnecchi et al. (2014) found a positive correlation between cognitive abilities and global resting-state network efficiency, characterized by weak levels of connectivity linking distant brain lobes between and within hemispheres. In addition, the functional meaning of high beta in the EEG literature has been related to anxiety and a “busy” brain (Thompson and Thompson, 2006). Therefore, a tentative hypothesis for these lower levels of connectivity in the beta 2 band as a reflection of high levels of neural efficiency can be related to low levels of anxiety and a more regulated brain, which in turn, performs cognitively better.

Moderation analyses were also conducted using each of the five CANTAB subscales as dependent variables instead of the composite score. The obtained results were somehow confirmatory of the general results. However, the CANTAB subscales are highly correlated, so the results should be taken cautiously. In detail, the analyses for the Spatial Span test (SSP), a measure of recent memory, showed that occipital delta CSD moderated the relationship between age and SSP performance: as occipital delta CSD increases, the age-related decline for SSP performance also increases, just like the results observed for the CANTAB composite score. Besides this confirmatory result, the interhemispheric LLC for temporal beta 1 and the right intrahemispheric LLC between frontal and occipital regions for beta 1 were also significant moderators of this relationship, with greater connectivity associated with a lower age-related decline in cognition. These results, again, point to the potential existence of compensatory mechanisms that are reflected in increased connectivity in high- than low-CR individuals (see Fleck et al., 2017, 2019 for similar interpretations).

In addition, for the Spatial Working Memory test (SWM), a measure of executive function, moderation results were the same as for the composite CANTAB score. Therefore, a more negative effect of age on executive function was observed as the beta 2 intrahemispheric LLC increased. Taken together, results of the analyses with the different CANTAB subtests, may indicate the coexistence of compensatory mechanisms and increased neural efficiency processes in the resting-state brain electrical activity of high CR individuals, in line with the manuscript’s main results. Nevertheless, these results suggest that the performance in different cognitive domains is supported by distinct neural correlates of CR. In that vein, previous studies have shown that CR may be associated with specific cognitive domains while unrelated to other domains (Lavrencic et al., 2018; Rodriguez et al., 2019) depending on the selected CR proxies, the cognitive tasks employed (Darby et al., 2017) or the individual experiences (Sandry et al., 2016). Therefore, future studies are warranted to deepen the understanding of the observed neural correlates of CR and their relationship with different cognitive domains assessed through several tasks.

Finally, it is important to highlight the absence of moderation effects on the relationship between age and cognitive function by the CRI measures (total index, Education, Working activity, and Leisure subindices). To consider them reliable correlates of CR, they would be expected to moderate the age and cognitive function relationship. One reason that could explain this discrepancy is that these indices may reflect the amount of CR acquired during a person’s lifetime (Nucci et al., 2012) but not the CR itself. This also happens with other socio-behavioral proxies of CR, such as education. Thus, the effect of these cumulative factors would be finally translated into more efficient neural processing and brain networks, reflecting the objective pathways of CR. Consequently, as proposed by Steffener and Stern (2012), CR may be supported by more adaptable functional brain processes that constitute a more direct and objective measure of CR than socio-behavioral indexes. Further, following a life span approach including young adults in the sample may have obscured the moderation effect that these indices may have between age and cognition when studied in older populations alone, given the CR is still building up. Therefore, the rsEEG markers observed in this study may be considered a more sensitive measure of CR-related neural changes and flexibility than paper and pencil measures such as the CRI.

In sum, many studies have proposed different neural measures as neural correlates of CR; however, as far as we know, none of them have tested the rsEEG variables as moderators in the relationship between age and cognitive status. Most of them have used the rsEEG measures as independent variables and split the sample into age and CR-based groups to perform ANOVAs. This approach presents limitations stemming from the use of an arbitrary median score to build the CR groups. Unlike previous studies, the strategy of the current work was not to compare age groups but instead to take the factor of age as a continuous measure. This life span approach, including participants from 18 to 82 years old, with higher ages indicating possible higher physiological neural deterioration, allows us to establish which rsEEG variables may be taken as probable protective or risk markers for accelerated cognitive decline as age increases. This, in turn, justifies to some extent the absence of a structural measure of brain integrity.

Moreover, this study followed a correlational and regressional approach and considered CR as the ability to recruit brain networks more efficiently. In light of the reviewed evidence, CR might be reflected by increases or decreases in activity and functional connections across the brain. Indeed, current results showed that as age increases, higher levels of occipital delta CSD and stronger LLC between medial and posterior regions for beta 2 may indicate a less effective way to recruit networks in people with lower levels of CR.

Although this results add valuable information to the research of aging and CR, they are not free from limitations and shortcomings. First, the large number of studied variables in this study increases the probability of a type I error, and, as such, some of our results could be false positives. However, given the study’s exploratory nature, which aims to generate hypotheses to be systematically and thoroughly evaluated in future studies, we adopted a rather lenient strategy regarding the problem of multiple comparisons. Second, there was a considerable amount of missing data for a dependent variable (i.e., CANTAB), which was subjected to a multiple imputation procedure. Although this is not the most desirable situation, a statistical comparison of CANTAB subtests’ score between the sample with imputed values and the sample without imputed values showed no significant differences, thus ensuring that the variable distributions were similar before and after the imputation procedure. Third, the lack of a measure of brain status did not allow us to align our results with the current research recommendations in the field of cognitive reserve (Stern et al., 2020). Hence, cross-sectional nature and lack of control of the study over these brain status variables make it possible that some results were influenced by factors such as brain amyloid burden, differences in brain volume, and the presence of genetic vulnerabilities. So, the insight into CR is somewhat limited. However, we have tried to minimize this limitation by taking a life span perspective and considering age as a risk factor for brain changes in order to be able to suggest hypotheses and guide subsequent studies. Fourth, the sample size may not be enough to draw firm conclusions, and more middle-aged participants should be included in future studies to make the age range smoother. Another possible shortcoming of the sample is that it is primarily female, which can impose limitations on the generalizability of the presented results. It is well-known that there are differences between women and men in brain aging, and there have also been previous findings on gender differences in CR studies (see, for example, Fleck et al., 2019). However, in this study, the variable sex was not a significant covariate in the moderation analyses. Finally, the lack of a defined and homogeneous protocol to study rsEEG and CR make the different studies not highly comparable since several methodological differences arose: the type of proxy measure used to obtain an estimation of CR, the different ways to calculate band amplitude or power and connectivity, the construction of the ROI(s) for the analyses, the statistical strategies, etc.

## Conclusion

In this study, we aimed to find possible neural correlates of CR that can be used as objective measures of CR instead of indirect proxies, such as educational attainment or intelligence measures. To this end, we analyzed dozens of measures of rsEEG, including eLORETA solutions for cortical current density sources and connectivity, as moderators on age-related changes in cognitive function. Moderation analyses showed that lower delta activity in the occipital region and lower connectivity of beta 2 in the right hemisphere between occipital and limbic regions could index compensatory mechanisms and increase neural efficiency to maintain high cognitive performance during the aging process. Therefore, these patterns of rsEEG activity might be considered as putative neural correlates of the cognitive reserve to be deeply studied with more statistical control. As stated before, this study is highly exploratory with many variables under study, aiming to generate hypotheses to be refuted in future projects. This type of objective neural measure could be useful in the future to prevent the appearance of cognitive impairment, allocating more preventive efforts to those adults with rsEEG markers of low CR.

## Data availability statement

The datasets presented in this study can be found in online repositories. The names of the repository/repositories and accession number(s) can be found below: Open Science Framework: https://osf.io/bdfyu/.

## Ethics statement

This study involves human participants, so its protocols were reviewed and approved by the Institutional Review Board of the University of Minho (CE.CVS 095/2018). The patients/participants provided their written informed consent to participate in this study.

## Author contributions

AB, AS, and DP involved in the conception and design of the original study, as well as in the enrolment of the participants, and controlled the interpretation of the results. AB and DP carried out the neuropsychological assessments and EEG recordings and performed the data analysis in terms of database construction, EEG data processing, and statistical analyses. AB generated the first draft of the manuscript, and developed the final version of the manuscript. AS and DP critically reviewed the draft and modified it accordingly. All authors contributed to the article and approved the submitted version.

## Footnotes

1
https://github.com/sccn/clean_rawdata

2 Any data value was considered an outlier if it lies outside of the following ranges: • 3rd quartile + 3*interquartile range. • 1st quartile − 3*interquartile range.
