# Supplementary material for: Resting-state electroencephalographic correlates of cognitive reserve: Moderating the age-related worsening in cognitive function
Source: Front Aging Neurosci. 2022 Sep 14;14:854928. doi: 10.3389/fnagi.2022.854928 (PMC9521492; doi:10.3389/fnagi.2022.854928)
Supplement: Supplementary file 1 [file Data_Sheet_1.docx]

***Supplementary Material***

**Supplementary Table 1.** Pearson's correlation coefficients and *p*-values for the relationship between Age and demographic and cognitive variables (n = 56).

|  | **Age** |  |
| --- | --- | --- |
| **Education years** | Pearson's correlation | **-0.637** |
|  | Sig. (bilateral) | **0.000** |
| **CRI Education** | Pearson's correlation | -0.041 |
|  | Sig. (bilateral) | 0.766 |
| **CRI Laboural** | Pearson's correlation | **0.402** |
|  | Sig. (bilateral) | **0.002** |
| **CRI Leisure** | Pearson's correlation | **0.305** |
|  | Sig. (bilateral) | **0.022** |
| **Total CRI** | Pearson's correlation | 0.235 |
|  | Sig. (bilateral) | 0.082 |
| **CANTAB RTI** | Pearson's correlation | **0.347** |
|  | Sig. (bilateral) | **0.009** |
| **CANTAB PAL** | Pearson's correlation | **0.703** |
|  | Sig. (bilateral) | **0.000** |
| **CANTAB SSP** | Pearson's correlation | **-0.638** |
|  | Sig. (bilateral) | **0.000** |
| **CANTAB MMT** | Pearson's correlation | **0.265** |
|  | Sig. (bilateral) | **0.048** |
| **CANTAB SWM** | Pearson's correlation | **0.600** |
|  | Sig. (bilateral) | **0.000** |
| **CANTAB composite** | Pearson's correlation | **-0.723** |
|  | Sig. (bilateral) | **0.000** |

**Supplementary Table 2.** Statistics for non-significant moderation models

| **rsEEG moderator** | **Statistics** |
| --- | --- |
| **Beta 2 occipital CSD** | **Model Summary**  R R-sq MSE F df1 df2 p  0.7313 0.5347 0.2501 14.6539 4.0000 51.0000 0.0000  **Model**  Coeff se t p LLCI ULCI  constant -0.0636 0.2437 -0.2610 0.7952 -0.5529 0.4257  Age -0.0211 0.0028 -7.5092 0.0000 -0.0267 -0.0154  B2O_CSD -0.2619 0.4860 -0.5388 0.5924 -1.2377 0.7139  Int_1 -0.0156 0.0186 -0.8413 0.4041 -0.0529 0.0216  Sex 0.0542 0.2085 0.2598 0.7961 -0.3644 0.4727  **Product terms key:**  Int_1 : Age x B2O_CSD  **Test(s) of highest order unconditional interaction(s):**  R2-chng F df1 df2 p  X*W 0.0065 0.7078 1.0000 51.0000 0.4041 |
| **Beta 1 temporal inter-hemispherical LLC** | **Model Summary**  R R-sq MSE F df1 df2 p  0.7358 0.5414 0.2465 15.0503 4.0000 51.0000 0.0000  **Model**  Coeff se t p LLCI ULCI  constant -0.1359 0.2391 -0.5683 0.5724 -0.6158 0.3441  Age -0.0205 0.0028 -7.2774 0.0000 -0.0262 -0.0149  B1T_LLC -0.2064 0.2063 -1.0004 0.3219 -0.6206 0.2078  Int_1 0.0090 0.0088 1.0233 0.3110 -0.0087 0.0268  Sex 0.1118 0.2033 0.5496 0.5850 -0.2965 0.5200  **Product terms key:**  Int_1 : Age x B1T_LLC  **Test(s) of highest order unconditional interaction(s):**  R2-chng F df1 df2 p  X*W 0.0094 1.0471 1.0000 51.0000 0.3110 |
| **Beta 1 occipital inter-hemispherical LLC** | **Model Summary**  R R-sq MSE F df1 df2 p  0.7274 0.5291 0.2531 14.3247 4.0000 51.0000 0.0000  **Model**  Coeff se t p LLCI ULCI  constant -0.0768 0.2457 -0.3123 0.7561 -0.5701 0.4166  Age -0.0216 0.0029 -7.4538 0.0000 -0.0274 -0.0158  B1O_LLC 0.0919 0.1776 0.5176 0.6070 -0.2646 0.4485  Int_1 -0.0046 0.0083 -0.5504 0.5844 -0.0212 0.0121  Sex 0.0739 0.2082 0.3549 0.7241 -0.3441 0.4919  **Product terms key:**  Int_1 : Age x B1O_LLC  **Test(s) of highest order unconditional interaction(s):**  R2-chng F df1 df2 p  X*W 0.0028 0.3030 1.0000 51.0000 0.5844 |
| **Right delta parietal-limbic intra-hemispherical LLC** | **Model Summary**  R R-sq MSE F df1 df2 p  0.7335 0.5380 0.2483 14.8480 4.0000 51.0000 0.0000  **Model**  Coeff se t p LLCI ULCI  constant -0.0650 0.2403 -0.2705 0.7878 -0.5475 0.4175  Age -0.0209 0.0029 -7.1181 0.0000 -0.0268 -0.0150  RDPL_LLC 0.2052 0.3011 0.6815 0.4986 -0.3993 0.8096  Int_1 0.0087 0.0122 0.7150 0.4779 -0.0158 0.0332  Sex 0.0686 0.2039 0.3365 0.7379 -0.3408 0.4781  **Product terms key:**  Int_1 : Age x RDPL_LLC  **Test(s) of highest order unconditional interaction(s):**  R2-chng F df1 df2 p  X*W 0.0046 0.5112 1.0000 51.0000 0.4779 |
| **Right beta 1 frontal-occipital intra-hemispherical LLC** | **Model Summary**  R R-sq MSE F df1 df2 p  0.7400 0.5476 0.2432 15.4324 4.0000 51.0000 0.0000  **Model**  Coeff se t p LLCI ULCI  constant -0.1821 0.2460 -0.7403 0.4625 -0.6759 0.3117  Age -0.0212 0.0028 -7.6227 0.0000 -0.0267 -0.0156  RB1FO_LLC 0.0786 0.1974 0.3982 0.6921 -0.3177 0.4749  Int_1 0.0127 0.0088 1.4459 0.1543 -0.0049 0.0304  Sex 0.1508 0.2086 0.7230 0.4730 -0.2680 0.5696  **Product terms key:**  Int_1 : Age x RB1FO_LLC  **Test(s) of highest order unconditional interaction(s):**  R2-chng F df1 df2 p  X*W 0.0185 2.0905 1.0000 51.0000 0.1543 |
| **Right beta 1 central-occipital intra-hemispherical LLC** | **Model Summary**  R R-sq MSE F df1 df2 p  0.7305 0.5336 0.2507 14.5862 4.0000 51.0000 0.0000  **Model**  Coeff se t p LLCI ULCI  constant -0.0408 0.2470 -0.1653 0.8693 -0.5367 0.4550  Age -0.0215 0.0028 -7.5797 0.0000 -0.0272 -0.0158  RB1CO_LLC 0.0776 0.2066 0.3758 0.7087 -0.3372 0.4925  Int_1 -0.0090 0.0095 -0.9457 0.3487 -0.0280 0.0100  Sex 0.0414 0.2100 0.1973 0.8443 -0.3802 0.4631  **Product terms key:**  Int_1 : Age x RB1CO_LLC  **Test(s) of highest order unconditional interaction(s):**  R2-chng F df1 df2 p  X*W 0.0082 0.8944 1.0000 51.0000 0.3487 |
| **Left alpha 1 frontal-temporal intra-hemispherical LLC** | **Model Summary**  R R-sq MSE F df1 df2 p  0.7500 0.5625 0.2352 16.3902 4.0000 51.0000 0.0000  **Model**  Coeff se t p LLCI ULCI  constant -0.1513 0.2333 -0.6486 0.5195 -0.6198 0.3171  Age -0.0216 0.0027 -7.9069 0.0000 -0.0271 -0.0161  LA1FT_LLC 0.0319 0.1766 0.1806 0.8574 -0.3227 0.3865  Int_1 0.0128 0.0071 1.8085 0.0764 -0.0014 0.0270  Sex 0.1211 0.1985 0.6099 0.5446 -0.2775 0.5196  **Product terms key:**  Int_1 : Age x LA1FT_LLC  **Test(s) of highest order unconditional interaction(s):**  R2-chng F df1 df2 p  X*W 0.0281 3.2708 1.0000 51.0000 0.0764 |
| **Left beta 1 frontal-temporal intra-hemispherical LLC** | **Model Summary**  R R-sq MSE F df1 df2 p  0.7294 0.5320 0.2515 14.4961 4.0000 51.0000 0.0000  **Model**  Coeff se t p LLCI ULCI  constant -0.1275 0.2420 -0.5267 0.6007 -0.6133 0.3584  Age -0.0211 0.0028 -7.4353 0.0000 -0.0269 -0.0154  LB1FT_LLC 0.1090 0.2272 0.4799 0.6334 -0.3470 0.5650  Int_1 0.0083 0.0101 0.8184 0.4170 -0.0120 0.0285  Sex 0.1053 0.2057 0.5120 0.6109 -0.3076 0.5183  **Product terms key:**  Int_1 : Age x LB1FT_LLC  **Test(s) of highest order unconditional interaction(s):**  R2-chng F df1 df2 p  X*W 0.0061 0.6697 1.0000 51.0000 0.4170 |
| **Left beta 1 frontal-occipital intra-hemispherical LLC** | **Model Summary**  R R-sq MSE F df1 df2 p  0.7409 0.5489 0.2425 15.5138 4.0000 51.0000 0.0000  **Model**  Coeff se t p LLCI ULCI  constant -0.1576 0.2387 -0.6601 0.5121 -0.6368 0.3217  Age -0.0214 0.0028 -7.7376 0.0000 -0.0270 -0.0159  LB1FO_LLC 0.2645 0.2009 1.3166 0.1939 -0.1388 0.6677  Int_1 0.0078 0.0093 0.8382 0.4058 -0.0108 0.0264  Sex 0.1359 0.2033 0.6686 0.5068 -0.2722 0.5441  **Product terms key:**  Int_1 : Age x LB1FO_LLC  **Test(s) of highest order unconditional interaction(s):**  R2-chng F df1 df2 p  X*W 0.0062 0.7026 1.0000 51.0000 0.4058 |
| **Left beta 1 parietal-temporal intra-hemispherical LLC** | **Model Summary**  R R-sq MSE F df1 df2 p  0.7389 0.5459 0.2441 15.3275 4.0000 51.0000 0.0000  **Model**  Coeff se t p LLCI ULCI  constant -0.1654 0.2437 -0.6785 0.5005 -0.6547 0.3240  Age -0.0213 0.0028 -7.6376 0.0000 -0.0269 -0.0157  LB1PT_LLC 0.1630 0.2025 0.8049 0.4246 -0.2436 0.5696  Int_1 0.0123 0.0095 1.2874 0.2038 -0.0069 0.0314  Sex 0.1355 0.2065 0.6560 0.5148 -0.2791 0.5500  **Product terms key:**  Int_1 : Age x LB1PT_LLC  **Test(s) of highest order unconditional interaction(s):**  R2-chng F df1 df2 p  X*W 0.0148 1.6573 1.0000 51.0000 0.2038 |
| **Left beta 1 temporal-occipital intra-hemispherical LLC** | **Model Summary**  R R-sq MSE F df1 df2 p  0.7264 0.5276 0.2539 14.2399 4.0000 51.0000 0.0000  **Model**  Coeff se t p LLCI ULCI  constant -0.1000 0.2487 -0.4023 0.6892 -0.5993 0.3992  Age -0.0214 0.0029 -7.3988 0.0000 -0.0273 -0.0156  LB1TO_LLC 0.1047 0.2034 0.5146 0.6090 -0.3037 0.5131  Int_1 -0.0004 0.0096 -0.0433 0.9656 -0.0197 0.0189  Sex 0.0895 0.2100 0.4260 0.6719 -0.3321 0.5110  **Product terms key:**  Int_1 : Age x LB1TO_LLC  **Test(s) of highest order unconditional interaction(s):**  R2-chng F df1 df2 p  X*W 0.0000 0.0019 1.0000 51.0000 0.9656 |

**Supplementary Table 3.** Statistics for moderation models with CRI measures (total CRI, CRI-Education, CRI-Working Activity and CRI-Leisure) as moderators in the relationship between age and cognitive status.

| **CRI Moderator** | **Statistics** |
| --- | --- |
| **Total CRI** | **Model Summary**  R R-sq MSE F df1 df2 p  0.7343 0.5393 0.2477 14.9226 4.0000 51.0000 0.0000  **Model**  Coeff se t p LLCI ULCI  constant -0.0129 0.2512 -0.0515 0.9591 -0.5173 0.4914  Age -0.0220 0.0030 -7.2645 0.0000 -0.0281 -0.0159  CRI 0.0012 0.0058 0.2109 0.8338 -0.0104 0.0128  Int_1 -0.0003 0.0002 -1.1569 0.2527 -0.0008 0.0002  Sex 0.0316 0.2103 0.1505 0.8810 -0.3905 0.4537  **Product terms key:**  Int_1 : Age x CRI  **Test(s) of highest order unconditional interaction(s**):  R2-chng F df1 df2 p  X*W 0.0121 1.3384 1.0000 51.0000 0.2527 |
| **CRI Education** | **Model Summary**  R R-sq MSE F df1 df2 p  0.7333 0.5377 0.2485 14.8279 4.0000 51.0000 0.0000  **Model**  Coeff se t p LLCI ULCI  constant -0.0189 0.2479 -0.0762 0.9395 -0.5166 0.4788  Age -0.0214 0.0028 -7.5858 0.0000 -0.0271 -0.0157  CRI_Edu 0.0028 0.0037 0.7636 0.4486 -0.0046 0.0103  Int_1 -0.0002 0.0002 -0.9679 0.3377 -0.0005 0.0002  Sex 0.0141 0.2129 0.0662 0.9475 -0.4133 0.4415  **Product terms key:**  Int_1 : Age x CRI_Edu  **Test(s) of highest order unconditional interaction(s):**  R2-chng F df1 df2 p  X*W 0.0085 0.9368 1.0000 51.0000 0.3377 |
| **CRI Working activity** | **Model Summary**  R R-sq MSE F df1 df2 p  0.7490 0.5610 0.2359 16.2954 4.0000 51.0000 0.0000  **Model**  Coeff se t p LLCI ULCI  constant -0.0651 0.2342 -0.2779 0.7822 -0.5352 0.4050  Age -0.0184 0.0039 -4.7421 0.0000 -0.0262 -0.0106  CRI_Lab -0.0128 0.0101 -1.2712 0.2094 -0.0331 0.0074  Int_1 0.0000 0.0004 0.1046 0.9171 -0.0008 0.0009  Sex 0.0532 0.2000 0.2661 0.7913 -0.3484 0.4548  **Product terms key:**  Int_1 : Age x CRI_WA  **Test(s) of highest order unconditional interaction(s):**  R2-chng F df1 df2 p  X*W 0.0001 0.0109 1.0000 51.0000 0.9171 |
| **CRI Leisure** | **Model Summary**  R R-sq MSE F df1 df2 p  0.7255 0.5264 0.2546 14.1705 4.0000 51.0000 0.0000  **Model**  Coeff se t p LLCI ULCI  constant -0.0788 0.2598 -0.3034 0.7628 -0.6004 0.4427  Age -0.0211 0.0037 -5.7857 0.0000 -0.0285 -0.0138  CRI_Lei -0.0007 0.0118 -0.0631 0.9499 -0.0245 0.0230  Int_1 -0.0001 0.0005 -0.1369 0.8917 -0.0010 0.0009  Sex 0.0750 0.2121 0.3537 0.7250 -0.3508 0.5008  **Product terms key:**  Int_1 : Age x CRI_Lei  **Test(s) of highest order unconditional interaction(s):**  R2-chng F df1 df2 p  X*W 0.0002 0.0187 1.0000 51.0000 0.8917 |

**Supplementary Table 4.** Statistics for significant interaction terms in moderation analyses using CANTAB sub-scales as dependent variables.

***4.1. CANTAB SSP***

| **rsEEG moderator** | **Statistics** |
| --- | --- |
| **Delta occipital CSD** | **Model Summary**  R R-sq MSE F df1 df2 p  0.6958 0.4841 1.5388 11.9643 4.0000 51.0000 0.0000  **Model**  Coeff se t p LLCI ULCI  constant 4.7568 0.6116 7.7777 0.0000 3.5290 5.9846  Age -0.0438 0.0074 -5.9226 0.0000 -0.0586 -0.0290  DelO_CSD 0.1193 0.9188 0.1299 0.8972 -1.7252 1.9638  Int_1 -0.0902 0.0389 -2.3212 0.0243 -0.1683 -0.0122  Sex 0.8737 0.5153 1.6953 0.0961 -0.1609 1.9082  **Product terms key:**  Int_1 : Age x DelO_CSD  **Test(s) of highest order unconditional interaction(s):**  R2-chng F df1 df2 p  X*W 0.0545 5.3879 1.0000 51.0000 0.0243 |
| **Beta 1 temporal inter-hemispherical LLC** | **Model Summary**  R R-sq MSE F df1 df2 p  0.6979 0.4871 1.5300 12.1074 4.0000 51.0000 0.0000  **Model**  Coeff se t p LLCI ULCI  constant 4.8502 0.5956 8.1429 0.0000 3.6544 6.0459  Age -0.0424 0.0070 -6.0361 0.0000 -0.0565 -0.0283  B1T _LLC -0.6941 0.5140 -1.3505 0.1828 -1.7259 0.3377  Int_1 0.0471 0.0220 2.1395 0.0372 0.0029 0.0913  Sex 0.8640 0.5066 1.7057 0.0941 -0.1529 1.8810  **Product terms key:**  Int_1 : Age x B1T_LLC  **Test(s) of highest order unconditional interaction(s):**  R2-chng F df1 df2 p  X*W 0.0460 4.5773 1.0000 51.0000 0.0372 |
| **Right beta 1 frontal-occipital intra-hemispherical LLC** | **Model Summary**  R R-sq MSE F df1 df2 p  0.6885 0.4740 1.5691 11.4883 4.0000 51.0000 0.0000  **Model**  Coeff se t p LLCI ULCI  constant 4.6562 0.6248 7.4522 0.0000 3.4018 5.9105  Age -0.0442 0.0071 -6.2592 0.0000 -0.0583 -0.0300  RB1FO_LLC -0.3238 0.5014 -0.6458 0.5213 -1.3305 0.6828  Int_1 0.0460 0.0223 2.0575 0.0448 0.0011 0.0908  Sex 1.0436 0.5299 1.9693 0.0544 -0.0203 2.1075  **Product terms key:**  Int_1 : Age x RB1RFO_LLC  **Test(s) of highest order unconditional interaction(s):**  R2-chng F df1 df2 p  X*W 0.0437 4.2331 1.0000 51.0000 0.0448 |

***4.2. CANTAB SWM***

| **rsEEG moderator** | **Statistics** |
| --- | --- |
| **Right beta 2 occipital-limbic intra-hemispherical LLC** | Model **Summary**  R R-sq MSE F df1 df2 p  0.6456 0.4168 58.9106 9.1109 4.0000 51.0000 0.0000  **Model**  Coeff se t p LLCI ULCI  constant 17.6200 3.7993 4.6377 0.0000 9.9926 25.2474  Age 0.2523 0.0447 5.6483 0.0000 0.1626 0.3420  RB2OL_LLC 1.8095 3.8362 0.4717 0.6391 -5.8920 9.5110  Int_1 0.3295 0.1650 1.9963 0.0513 -0.0019 0.6608  Sex -0.8166 3.1960 -0.2555 0.7994 -7.2328 5.5996  **Product terms key:**  Int_1 : Age x RB2OL_LLC  **Test(s) of highest order unconditional interaction(s):**  R2-chng F df1 df2 p  X*W 0.0456 3.9851 1.0000 51.0000 0.0513 |

**Supplementary information:**

1. **Gamma analysis and results**

Given the exploratory nature of the present study, we have performed gamma analysis in order to cover the whole frequencies spectra. The procedure was exactly the same as for the rest of frequency bands. The cortical sources of spectral density with a resolution of 0.5 Hz were estimated for a range between 30.5 to 40 Hz. Also, the lagged-linear connectivity (LLC) was calculated as a measure of interdependence of rsEEG sources.

Gamma rsEEG variables were transformed to a logarithmic scale (log-10). No outliers were detected. The association between the total CRI and the gamma rsEEG variables was tested through a series of bivariate correlations (6 CSD variables; 6 inter-hemispheric LLC variables; and 30 intra-hemispheric LLC variables). Finally, a moderation analysis using the regression-based approach in Hayes’ (2017) PROCESS macro (Version 3.5) for IBM SPSS was conducted (Model 1; see Figure 2), with Age as a continuous independent variable (X), CANTAB composite score as the dependent variable (Y), and the gamma rsEEG variables with a moderate correlation with the total CRI as moderator variables. Sex was included as a covariate.

Just one gamma variable showed a moderate effect size correlation with total CRI: the intra-hemispheric LLC involving parietal and temporal regions in the right hemisphere (Pearson's correlation = 0.32; 95 % CI = 0.07-0.51). However, this variable did not moderate the relationship between age and cognitive status, being the interaction term between age and intra-hemispheric LLC between parietal and temporal regions for gamma activity in the right hemisphere not significant, *b* = -0.004, *t*(51) = -0.38, BootLLCI = -0.023-BootULCI = 0.016; *p* = 0.71; *ΔR^2^* = 0.001, *F*(1,51) = 0.144.

This moderate correlation is partially in agreement with previous gamma results related to cognitive reserve. For example, Fleck et al. (2017) found greater connectivity for gamma band in high-CR than in low-CR participants but just for older adults, not for the younger participants in their study. Similarly, Yang and Lin (2020) found higher resting-state gamma intensity in the right temporal region for participants with high than low CR, in a MEG study. These authors interpreted their findings as evidence for better neurocognitive activities such as perception and working memory in healthy aging (Yang & Lin, 2020). Moreover, the higher levels of gamma activity in the right hemisphere for older adults were interpreted as a mechanism for neural compensation in high-CR (Fleck et al., 2017).

1. **Multiple Imputation procedure**

The APA Task Force on Statistical Inference explicitly warned against the use of simple methods of imputation such as listwise or pairwise deletion (see Dong & Peng, 2013) when dealing with missing values. For multiple imputation procedures, instead of filling in a single value for each missing value, a set of plausible values that represent the uncertainty about the right value to impute are produced for each missing value. This process results in valid statistical inferences that properly reflect the uncertainty due to missing values (Yuan, 2000). Predictive mean matching (PMM), the specific multiple imputation procedure used in the current study is now one of the most accepted methods for multiple imputation among researchers in social sciences due to its robustness and bias-free estimates. It is a hot deck procedure, which finds a suitable “donor” for a missing case by using similar (in some characteristics) observed cases. Thus, drawing the “donor” case from a pool. To do so, it matches the cases via the closeness of the predicted means (in a regression model). The “winner donor” in the pool is the one with predicted values closer to the predicted values for the missing case. We chose this procedure since it is less sensitive than parametric procedures to regression model misspecifications, such as nonlinear associations, heteroscedasticity, deviations from normality or when the assumptions for the regression model are unmet (Morris, White, & Royston, 2014). That way, PMM imputed data usually preserves the distribution of the observed data (see below). Furthermore, PMM imputations yield “valid” values, in the sense they will fit the respective scale of measurement for a given variable, and will always lie within the range of observed values. Additionally, a recent simulation study has shown it is a robust and bias-free procedure in samples as small as N = 20 even if combined with large amounts of missing data (40 - 50 %) (Kleinke, 2018).

So, given the observed percentage of missing data was within the range of those commonly encountered (15-20%) in educational and psychological studies (Enders, 2003), we decided to proceed with the multiple imputation.

First, in order to check that the missing data do not follow a defined pattern, which is a requisite for multiple imputation of missing data, we performed the Missing Completely at Random (MCAR) test of Little (Little, 1988). Little’s MCAR test results were non-significant; thus, we can assure that the missing values were completely at random. Further, to check that the multiple imputation process had worked properly, we compared the distribution of the variables from the original sample without imputations with the distribution of the same variables after the multiple imputation process. To that end, we conducted a one-way ANOVA, which pointed to the absence of significant mean differences between the two conditions for any of the CANTAB subscales:

- RTI (56 participants versus 45): means 298.12 versus 296.99; F(1) = 0.005, p = 0.95

- PAL (56 participants versus 45): means 17.13 versus 17.16; F(1) = 0.000, p =0.99

- SWM (56 participants versus 45): means 16.11 versus 16.11; F(1) = 0.000, p = 0.99

- SSP (56 participants versus 44): means 5.88 versus 5.86; F(1) = 0.001, p = 0.98

- MMT (56 participants versus 44): means 254.88 versus 254.85, F(1) = 0.000, p =0.99

**REFERENCES**

Dong, Y., & Peng, C. Y. J. (2013). Principled missing data methods for researchers. SpringerPlus, 2(1), 1-17

Enders, C. K. (2003). Using the Expectation Maximization Algorithm to Estimate Coefficient Alpha for Scales With Item-Level Missing Data. Psychol. Meth., 8(3), 322–337. doi: 10.1037/1082-989X.8.3.322

Fleck, J. I., Kuti, J., Mercurio, J., Mullen, S., Austin, K., and Pereira, O. (2017). The impact of age and cognitive reserve on resting-state brain connectivity. Front. Aging Neurosci. 9 :392. doi:10.3389/fnagi.2017.00392

Hayes, A. F. (2017). Introduction to mediation, moderation, and conditional process analysis: A regression-based approach. New York: Guilford Press.

Kleinke, K. (2018). Multiple imputation by predictive mean matching when sample size is small. Methodology 14, 3-15

Little, R. J. (1988). A test of missing completely at random for multivariate data with missing values. Journal of the American statistical Association, 83(404), 1198-1202.).

Morris, T. P., White, I. R and Royston, P. (2014). Tuning multiple imputation by predictive mean matching and local residual draws. BMC medical research methodology 14.1, 1-13

Yang, C. Y., and Lin, C. P. (2020). Classification of cognitive reserve in healthy older adults based on brain activity using support vector machine. Physiol. Meas. 41, 065009. doi: 10.1088/1361-6579/ab979e.

Yuan, Y. C. (2000). Multiple imputation for missing data: Concepts and new development. In Proceedings of the Twenty-Fifth Annual SAS Users Group International Conference (Vol. 267, No. 11
